# Establishment and transfer of classical eyeblink conditioning using electrical microstimulation of the hippocampus as the conditioned stimulus

**DOI:** 10.1371/journal.pone.0178502

**Published:** 2017-06-02

**Authors:** Juan Yao, Bing Wu, Guang-yan Wu, Xuan Li, Jian-ning Ye, Jian-feng Sui

**Affiliations:** 1 Department of Physiology, College of Basic Medical Science, Third Military Medical University, Chongqing, P.R. China; 2 Experimental Center of Basic Medicine, College of Basic Medical Science, Third Military Medical University, Chongqing, P.R. China; 3 Department of Neurology, Xinqiao Hospital, Third Military Medical University, Shapingba District, Chongqing, P.R. China; Tokai University, JAPAN

## Abstract

The present experiment was designed to determine whether classical eyeblink conditioning (EBC) can be established by using electrical microstimulation of the hippocampus as a conditioned stimulus (CS) paired with an air-puff unconditioned stimulus (US). We intended to examine whether EBC transfer could occur when a CS was shifted between microstimulation of the hippocampus as a CS (Hip-CS) and tone as a CS (tone-CS) and to compare the difference in transfer effectiveness between delay EBC (dEBC) and trace EBC (tEBC). Eight groups of guinea pigs, including 4 experimental groups and 4 control groups, were included in the study. First, the experimental groups received either a Hip-CS or a tone-CS paired with a US; then, these groups were exposed to a shifted CS (tone-CS or Hip-CS) paired with the US. The control groups received the corresponding Hip-CS or tone-CS, which was, however, pseudo-paired with the US. The control groups were then shifted to the tone-CS (or Hip-CS) paired with the US. The results show that EBC can be successfully established when using microstimulation of the hippocampus as a CS paired with an air-puff US, and that the acquisition rates of EBC are higher in the experimental groups than in the control groups after switching from the Hip-CS to the tone-CS or vice versa, indicating the occurrence of learning transfer between EBC established with the Hip-CS and tone-CS. The present study also demonstrated that the EBC re-acquisition rates were remarkably higher in dEBC than in tEBC with both types of transfer, which suggests that the saving effect was more evident in dEBC than tEBC. These results significantly expand our knowledge of EBC transfer as well as the functional neural circuit underlying EBC transfer.

## Introduction

For a long time, electrical microstimulation of specific brain regions has been used to provide information about the functions of brain structures related to specific behaviors. Establishment of a conditioned reflex with direct electrical stimulation of the cortical or subcortical brain regions as a conditioned stimulus (CS) was first developed and systematically investigated by Doty [1–4]. Grigoryan reported that direct electrical stimulation of the hippocampus can establish instrumental defensive conditioned reflexes in dogs, and after the establishment, the conditioned reflex can be generalized in response to test stimulation of several limbic structures [5]. Microstimulation of specific brain loci as CS has also been successfully used in the study of eyeblink conditioning (EBC), a widely used model for clarifying the neuronal mechanisms underlying associative learning and memory [6–8]. Numerous studies have shown that microstimulation of selected brain structures, such as the cochlear nucleus [9], medial auditory thalamic [10], auditory cortex [11], lateral geniculate, superior colliculus, visual cortex [12], pontine nuclei [13–16], cerebellar mossy fibers and parallel fibers [17–20], interpositus nucleus [21, 22], primary somatosensory cortex and coronal-precruciate cortex [23–25], and medial prefrontal cortex [26], can serve as effective CS to establish EBC [26]. However, whether microstimulation of the hippocampus is a sufficient CS to support EBC remains unknown.

Establishment of EBC requires the repeated paired presentation of a CS (usually a tone) with an unconditioned stimulus (US, e.g., a periorbital shock). The EBC protocol comprises the following two paradigms based on the temporal relationship between a CS and US: delay EBC (dEBC), where the onset of the CS precedes that of the US, but they coterminate, and trace EBC (tEBC) in which the CS and US are presented separately in time such that a stimulus-free period (trace interval) exists between the CS and US. It has been well established that both dEBC and tEBC are dependent on the cerebellum-brainstem circuit; however, tEBC requires additional modulations from structures outside the cerebellum, including the hippocampus and the mPFC [6, 27, 28]. Previous studies have shown that during training for the classically conditioned nictitating membrane (NM) response, the hippocampus shows an increase in unit firing that precedes the learned response [29, 30]. Thus, it is reasonable to postulate that functional connections exist between the hippocampus and EBC circuitry and that microstimulation of the hippocampus is a potentially sufficient CS to support EBC.

Leal-Campanario et al. [23] have reported that classical EBC can be established with electrical microstimulation of the primary somatosensory cortex as the CS (CS1) paired with a corneal air puff as the US. After the initial acquisition of EBC, they found that the EBC acquisition in response to the peripheral CS (CS2) developed at an accelerated rate compared to the control. The reverse experiment (shifting the CS from peripheral to central) revealed a similar result. It is believed that learning transfer results from the general transfer of the association between a CS and a US rather than the stimulation generalization [31, 32] and that it supports the multiple distributed characteristic of associative learning [23, 33].

To date, the characteristics and mechanisms of learning transfer remain unclear. Previous studies have demonstrated that learning transfer, a special type of cross-modal transfer, occurs when a CS is switched from stimulation of the primary sensory cortex to stimulation of the peripheral sensors [23]. Our previous study also showed that learning transfer of classical EBC can occur between electrical microstimulation of the mPFC and a tone as the CS in guinea pigs [34]. Still, little is known about the transfer effect when a CS is shifted from hippocampal stimulation (if it is a sufficient CS to support EBC) to peripheral stimulation or vice versa as well as the difference in learning transfer between dEBC and tEBC. Given the well-established facts that the hippocampus is engaged in tEBC but not in dEBC [35] and in recent tEBC but not in remote tEBC [36, 37], and that a well-known hippocampal time-limited role has also been found in a variety of memory tasks [38], the present study aimed to achieve the following goals: (1) to determine whether microstimulation of the hippocampus is a sufficient CS to support EBC; (2) to observe whether transfer of EBC learning can occur when a CS is shifted between microstimulation of the hippocampus and that of the peripheral stimulus; and (3) to compare the difference in transfer effectiveness between the two paradigms, dEBC and tEBC.

## Materials and methods (dx.doi.org/10.17504/protocols.io.hsib6ce)

### Subjects

A total of 48 adult male albino Dunkin-Hartley guinea pigs, weighing 500–600 g (4–5 months old) at the time of surgery, were included in the study. Before experiments and between conditioning sessions, these animals were individually housed in standard plastic cages on a 12:12 light/dark cycle with free access to food and water ad libitum. The room temperature was maintained at 23 ± 1°C. The procedures were approved by the Animal Care Committee of the Third Military Medical University and were performed in accordance with the principles outlined in the National Institutes of Health Guide for the Care and Use of Laboratory Animals.

### Surgery

**Fig 1:**
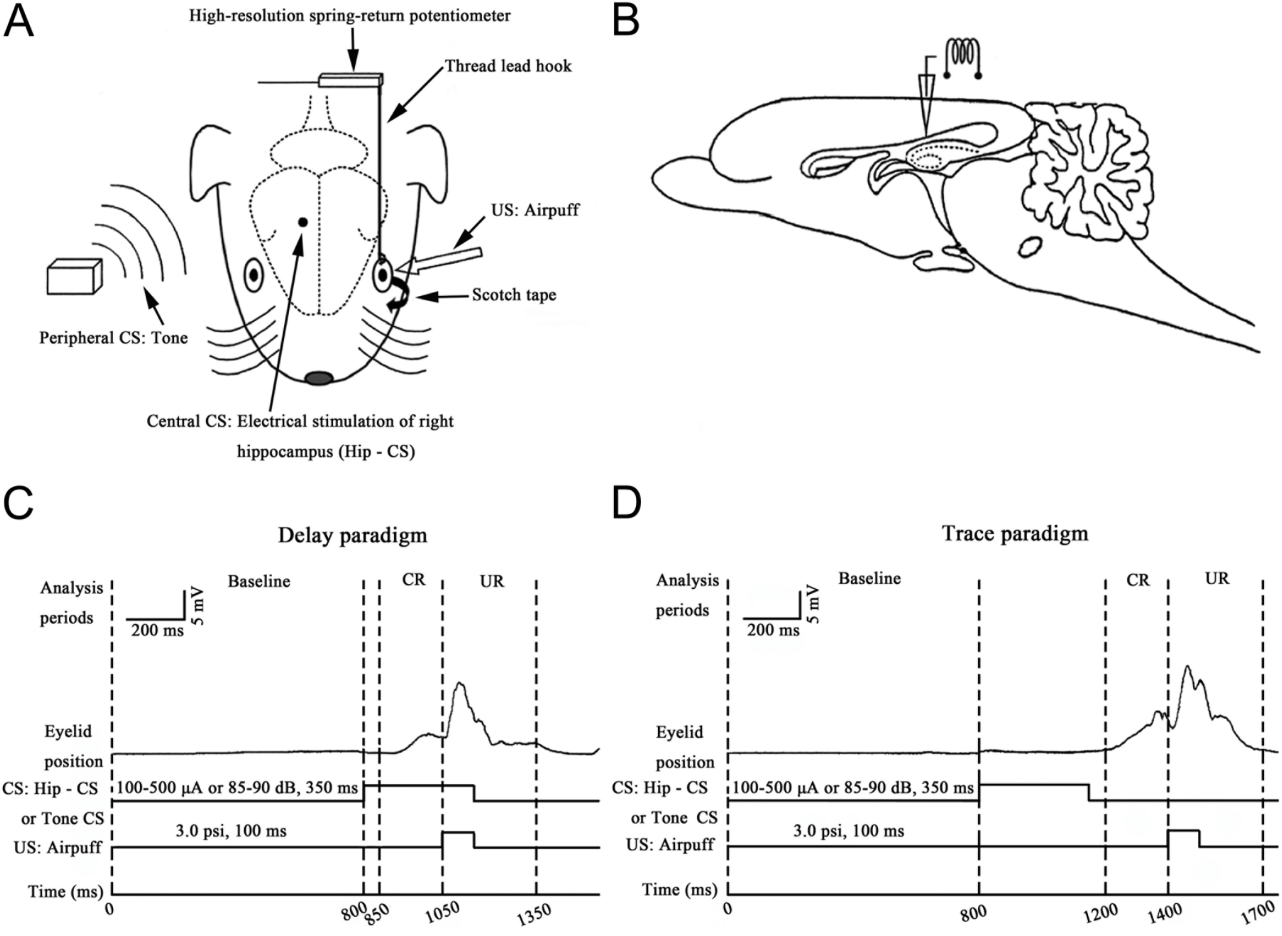
Experimental design. (A) Diagram of EBC measurement. The upper left eyelid movements were measured by a high-resolution spring-return potentiometer that was attached via a thread lead hooked with a nylon loop, which was sutured into the left upper eyelid. Bipolar electrodes were implanted in the right hippocampus (Hip). Electrical stimulation of right hippocampus (Hip-CS) or auditory stimulation (pure tone) was used as the conditioned stimulus (CS). Air-puff presented to the left cornea was used as the unconditioned stimulus (US). (B) Diagram of the sagittal section of a guinea pig brain, showing the electrical stimulation sites. (C, D) Schematic diagrams illustrate the temporal relationship between CS and US and analysis periods of CR and UR in delay (C) and trace (D) paradigms. In delay paradigm, US co-terminated with the offset of CS; and in trace paradigm, a stimulus-free trace interval of 250 ms was interposed between the CS offset and the US onset. The durations of CS and US were 350 ms and 100 ms, respectively. In each trial, parameters of the conditioned eyeblink response (CR; 50–250 ms period after the CS onset) and unconditioned eyeblink response (UR; 0–300 ms period after the US onset) were analyzed. These responses were defined based on the average magnitude of the baseline (a 0~800 ms period prior to the onset of the CS).

The surgical procedures for eyeblink recording were conducted essentially same as described by Yao et al. [34]. In brief, all animals were fitted with a headstage and a loop attached to the apex of the left upper eyelid. In the current study, this loop was utilized to attach the left upper eyelid to a movement-measuring device. The tension of the thread linking the eyelid loop and the transducer is so weak that this small resistance does not hinder the normal eyelid movement. Moreover, for each animal in every group, one small hole (diameter: 1.0 mm) was drilled on the right side of the skull centered on the right hippocampus at the following stereotaxic coordinates: anteroposterior (AP) +6.0 mm, mediolateral (ML) 5.0 mm relative to the frontal zero plane, and the midline sinus, respectively. Then, a bipolar stimulating electrode (No 792500, A-M Systems, Sequim, WA, USA; coated diameter: 332.00 μm, bare diameter: 254.00 μm) was implanted into the right hippocampus through the hole and the electrode’s tip was directed to the following stereotaxic coordinates: AP +6.0 mm, ML 5.0 mm, dorsoventral (DV) -4.5 mm to the skull surface (Fig 1A and 1B). To minimize animal suffering, all surgical interventions were carried out under satisfied surgical anesthesia with a mixture of ketamine (80mg/kg, i.p.) and xylazine (5mg/kg, i.p.). Other supportive veterinary care such as keep warm during operation and post-procedural analgesia by smearing the skin incision with 0.3 ml lidocanine solution (1.7%) once every 3 hours for the first 12 hours after operation, were also made to minimize potential distress or pain. The physical health of the animals were monitored and assessed twice a day by observing and measuring their locomotion, respiration, food-intake and mental conditions.

### Behavioral procedures

Animals were firstly adapted to the experimental environment for three sessions at 30 min per session, immediately followed by early training (or pseudo-training) sessions (stage I), transfer training sessions (stage II), and recall session (stage III). During these sessions, animals were restrained in a Plexiglas container (25 cm × 15 cm × 15 cm) located in a sound- and light-attenuated chamber, and their heads were secured with blunt ear bars that pressed on the head stages. The left eye of the animal was held open in a comfortable position, with the nylon loop sutured into the left upper eyelid, which was linked to the high-resolution potentiometer (JZ101, XH, Beijing, China). The voltage level represented the eyelid position, with baseline manually calibrated to a constant value. Moreover, the animal’s left lower eyelid was taped open. These measures ensured continual exposure of the animal’s left cornea.

The 48 male guinea pigs were divided into 8 groups, including 4 groups for study of delay paradigm (Fig 1C) and the other 4 for trace paradigm (Fig 1D). For both delay and trace paradigm studies, 2 groups (1 for experiment and 1 for control) were included for study of learning transfer from central to peripheral and another 2 for study of learning transfer from peripheral to central. In studies of learning transfer from central to peripheral (including for both dEBC and tEBC), animals firstly received electrical stimulation of the right hippocampus as CS (CS1, central or Hip-CS) and paired (for experimental group) or pseudo-paired (for control group) with corneal air-puff US, which lasting for 6 daily sessions in delay paradigms or 12 daily sessions in trace paradigms (stage I). Then, CS was switched from hippocampus stimulation to tone stimulation (CS2, peripheral or tone-CS) and paired with US (6 daily sessions for both experimental and control groups, stage Ⅱ). Finally, CS was shifted from peripheral to central again and paired with US (1 daily session, stage III) to test the EBC memory recall to CS1. In studies of learning transfer from peripheral to central, corresponding procedures were included as described above except the difference in CS patterns, i.e., CS1 was tone stimulation, and CS2 was hippocampus stimulation.

During behavior training with Hip-CS, the electrical constant current pulse train (350 ms duration) consisted of 70 pulses with single pulse width of 0.1 ms (cathodal, monophasic square, 200Hz) was repeatedly delivered via a stimulator (YC-2, Cheng Yi, Chengdu, China) and paired or pseudo-paired with the US. Current levels for electrical stimulation were adjusted to 40% of the minimum currents of eliciting measurable eyeblink responses [24], usually 100–300 μA for most animals. A binaural tone (2 kHz, 85–90 dB SPL, 5 ms rise/fall time) with duration of 350 ms was used as tone-CS, which was produced by a speaker placed 50 cm above the animal. A plastic pipe was placed 1.0 cm from the animal’s left eyeball for delivering a 100 ms duration air-puff (3.0 psi, measured at the end of pipe) to stimulate animal’s cornea and effect as US. Signals of eyelid-movement and CS/US were filtered with a bandpass of DC ~ 100 Hz or 0.1 ~ 3 kHz, respectively, digitized by a data-acquisition system (RM6280, Cheng Yi, Chengdu, China) at a sample rate of 10 kHz, and recorded simultaneously using the system’s built-in software (v 2.4). For delay paradigm in this study, the US co-terminated with the offset of the CS and a 250 ms delay interval between onsets of CS and of US emerged (Fig 1C); for trace paradigm, a stimulus-free trace interval of 250 ms was interposed between the CS offset and the US onset (Fig 1D). For the CS-US paired or pseudo-paired training, 60 trials were performed per day in a sound- and light- attenuated chamber, with inter-trial intervals varying randomly between 20 and 40 s. In the CS-US pseudo training paradigm, the US was presented at a random interval between 1 and 10 s after the CS onset.

### Histology

At the end of stage III, guinea pigs were deeply anesthetized with sodium pentobarbital (50 mg/kg, i.p.) and perfused transcardially with saline and 4% paraformaldehyde. The brains were removed and fixed in fresh paraformaldehyde solution for several days. Four days prior to sectioning, the brains were transferred to a 30% sucrose/4% paraformaldehyde solution. Frozen coronal sections at 30 μm in thickness were taken from the sites of the electrode implantation. The slices were stained with cresyl violet. The recognizable electrode tip tracks were examined carefully using a light microscope (SMZ1500, Nikon, Tokyo, Japan) with a digital camera (DXM1200F, Nikon, Tokyo, Japan) and were drawn onto plates using a stereotaxic atlas of the guinea pig brain [39]. Data from animals were excluded if the location of electrode tip could not be determined with a high degree of confidence.

### Behavioral data analysis

Each CS-US paired or pseudo-paired trial during recording was subdivided into three discontinuous analysis periods: (1) a “baseline” period, which occurred at 0–800 ms before the CS onset; (2) a “CR” period, which occurred at 200 ms before the US onset; and (3) a “UR” period, which occurred at 0–300 ms after the US onset (Fig 1C and 1D). A significant eyelid movement was defined as an increase in the mechanogram amplitude that was greater than the mean baseline amplitude, plus four times the standard deviation of the baseline activity. In addition, a significant eyelid movement was also required to have a minimal duration of 15 ms. Any significant eyelid movement during the latter two periods as defined above was counted as a CR or a UR, respectively. The percentage of CR (CR %) was defined as the ratio of the number of trials containing the CR to the total number of valid trials. To avoid disturbance in CR calculation from higher baseline noise, trials with sudden increased baseline signals of greater than the mean baseline amplitude plus four times the standard deviation of the baseline activity and lasted more than 15 ms will be excluded from analysis. Only trials with qualified baseline are defined as valid ones.

### Statistical analysis

All data were presented as means ± SEM. Statistical significance was determined by a least significant difference (LSD) post-hoc test following a two-way repeated measures analysis of variance (ANOVA), a separate one-way repeated measures ANOVA, or a separate one-way ANOVA using statistical software SPSS 18.0. A value of P < 0.05 was considered statistically significant for all tests.

## Results

### Electrode tip placements

**Fig 2:**
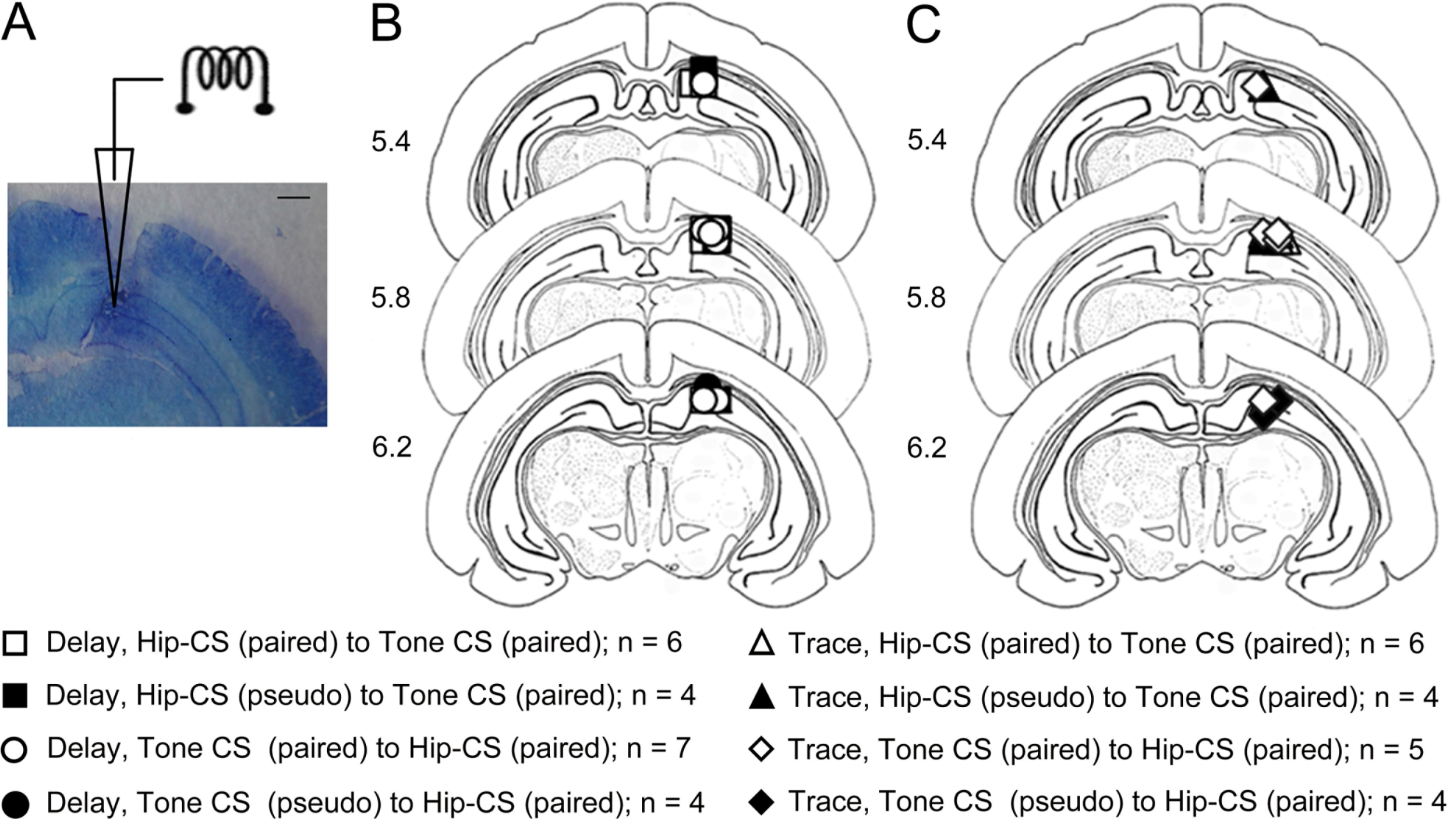
Locations of the electrode tips in the hippocampus of guinea pigs. (A) A representative of toluidine blue-stained coronal hippocampus section (30 μm) from a guinea pig that received hippocampal electrical stimulation as the CS. Scale bar represents 1.0 mm; (B, C) Schematic illustration of the locations of all electrode tips. (B) For delay paradigm: (□ group of from Hip-CS (paired) to tone-CS (paired), n = 6; ■ group of from Hip-CS (pseudo-paired) to tone-CS (paired), n = 4; ○ group of from tone-CS (paired) to Hip-CS (paired), n = 7; ● group of from tone-CS (pseudo-paired) to Hip-CS (paired), n = 4); (C) For trace paradigm: (△ group of from Hip-CS (paired) to tone-CS (paired), n = 6; ▲ group of from Hip-CS (pseudo-paired) to tone-CS (paired), n = 4; ◇ group of from tone-CS (paired) to Hip-CS (paired), n = 5; ◆ group of from tone-CS (pseudo-paired) to Hip-CS (paired), n = 4). The coronal brain plates are adapted from the atlas of Rapisarda and Bacchelli [39].

The locations of electrode tips were carefully checked before behavioral analysis. Data were excluded from the analysis if the electrode tip was not in the right location. As shown in Fig 2B and 2C, 40 of the 48 guinea pigs met our inclusion criteria, and their behavioral data were analyzed. In both delay (Fig 2B) and trace paradigms (Fig 2C), most of the electrode tips were placed in the right hippocampus (n = 21 for delay; n = 19 for trace paradigm) with 3 exceptions for the delay and 5 exceptions for the trace paradigm. Fig 2A is a representative photomicrograph of the electrode tips in the right hippocampus.

### After CS shift, EBC acquisition proceeds at a much faster rate in the groups that received paired CS1-US training in the first stage

**Fig 3:**
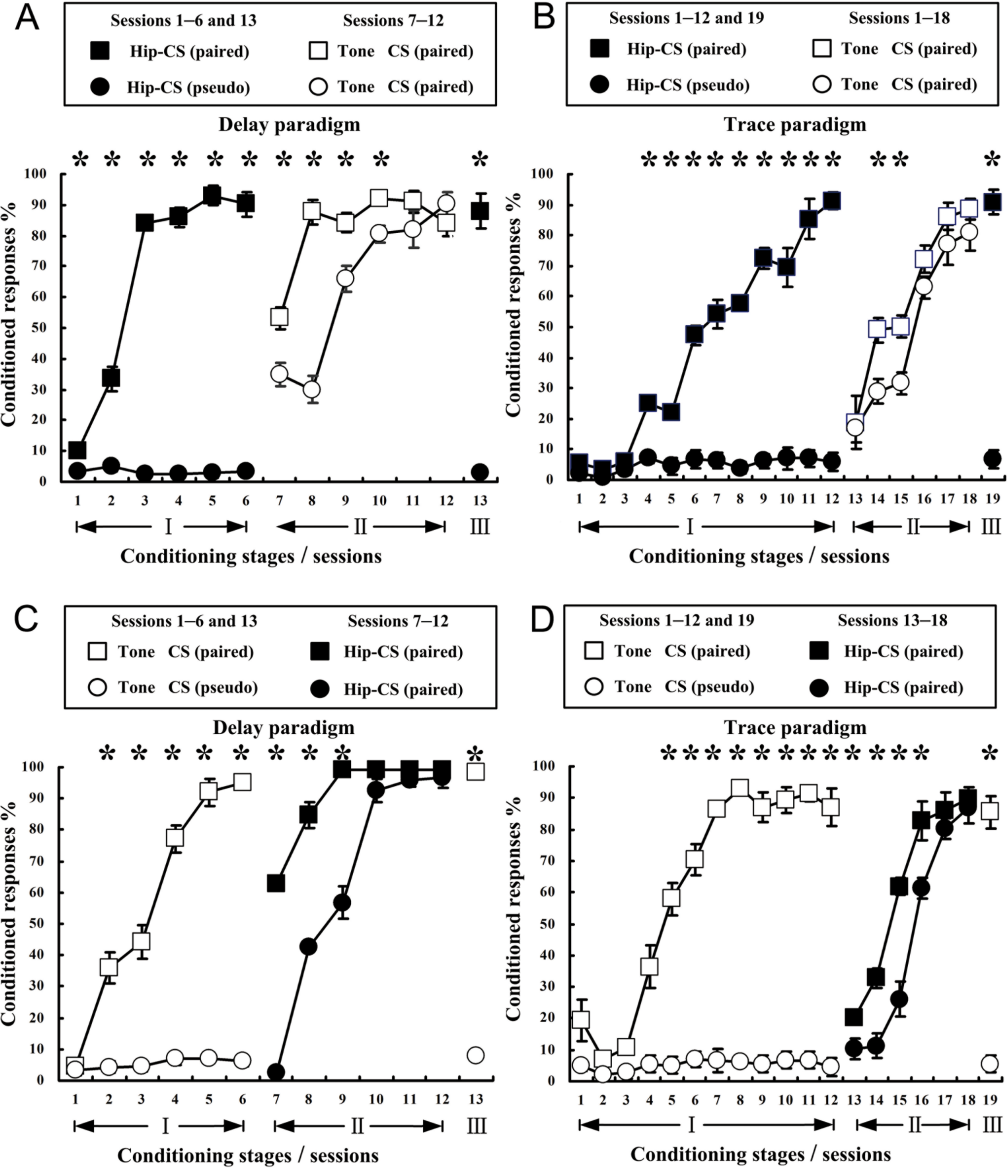
Acquisition curves of eyelid conditioned responses in delay and trace paradigms when CS shifted from central (Hip–CS) to peripheral (tone-CS) or vice versa. (A, B) Learning curves of dEBC (A) and tEBC (B) for groups of experiment (square, n = 6, for both dEBC and tEBC) and control (roundness, n = 4, for both dEBC and tEBC) when CS shifted from central to peripheral. Central CS (black in A, B) was presented during first 6 (dEBC) or 12 (tEBC) sessions in stage I and paired (black square in A and B) or pseudo-paired (black roundness in A, B) with US, then CS was switched to peripheral and paired with US (space square and space roundness, A and B) in sessions 7–12 (dEBC) or 13–18 (tEBC) of stage II. (C, D) Learning curves of dEBC (C) and tEBC (D) for groups of experiment (square, n = 7, for dEBC; n = 5, for tEBC) and control (roundness, n = 4, for both dEBC and tEBC) when CS shifted from peripheral to central (C, D). Central CS (space in C, D) was presented during first 6 (dEBC) or 12 (tEBC) sessions in stage I and paired (space square in C and D) or pseudo-paired (space roundness in C, D) with US, then CS was switched to central and paired with US (black square and black roundness, C, D) in sessions 7–12 (dEBC) or 13–18 (tEBC) of stage II. Data represent mean ± SEM. A two-way repeated measures ANOVA followed by the LSD post hoc test showed that there were significant differences in the percentages of the conditioned responses (CR) between groups of experiment and control in stage II in both delay and trace paradigms, either shifting CS from central to peripheral or vice versa. [Fig 3A and 3F (1, 8) = 40.028, *p < 0.05; Fig 3B and 3F (1, 8) = 8.905, *p < 0.05; Fig 3C and 3F (1, 9) = 154.691, *p < 0.05; Fig 3D and 3F (1, 7) = 16.299, *p < 0.05]. In recognition tests of stage III in the above 4 conditions, animals were all able to recall the original CR% to CS1.

Fig 3A and 3B illustrated the mean CR% of both dEBC and tEBC in experimental and control groups for learning transfer from central CS to peripheral CS. As shown in Fig 3A, the experimental animals successfully acquired Hip-CS-induced dEBC by the third session and maintained CR% stable in sessions 4–6 in stage I. In sessions 7–10 of stage II in which tone-CS was adopted, the experimental animals presented significantly more CRs than control animals who have experienced pseudo-paired training to central CS before CS shift. This result was confirmed by a two-way repeated measures ANOVA on the CR%, followed by the LSD post hoc test. There was a significant main group effect [F(1, 8) = 40.028, p < 0.001] in stage II. In the subsequent recognition test in the 13th session (stage III), animals who had experienced successive shift training from CS1 to CS2 could recall the original CR% to CS1. Fig 3B illustrated that experimental animals successfully acquired Hip-CS-induced tEBC by the 10th session in stage I. In session 14 and 15 of stage II in which tone-CS was used, the experimental animals presented significantly more CRs than control animals experienced pseudo-paired training to CS1. Similarly, the result was confirmed by a two-way repeated measures ANOVA on the CR%, followed by the LSD post hoc test. There was a significant main group effect [F(1, 8) = 8.905, p = 0.017] in stage II. The experimental animals could also recall the original CR% to CS1 in the recognition test in stage III.

Fig 3C and 3D demonstrated the mean CR% of both dEBC and tEBC in groups for study of learning transfer from peripheral to central. As displayed in Fig 3C, the experimental animals acquired a stable dEBC to tone-CS when paired with the US in stage I. In sessions 7–9 of stage II, the experimental animals displayed obviously more CRs than controls when CS was shifted from peripheral to central. A two-way repeated measures ANOVA on the CR% during stage II followed by the LSD post hoc test demonstrated a significant main group effect [F(1, 9) = 154.691, p < 0.001]. In the recognition test in the thirteenth session of stage III, the experimental animals were able to recall the original CR% to CS1. Fig 3D illustrated that after the acquisition of a stable tEBC to tone-CS in stage I, the experimental animals displayed remarkably more CRs than controls when CS was shifted from peripheral to central, in sessions 13–16 of stage II. A two-way repeated measures ANOVA on the CR% during stage II followed by the LSD post hoc test demonstrated a significant main group effect [F(1, 7) = 16.299, p = 0.005]. In the recognition test in session 19 of stage III, the experimental animals were able to recall the original trace CR% to CS1.

### Before CS shift, dEBC proceeds at a significantly faster rate than tEBC when cued with Hip-CS or tone-CS; Hip-CS-cued dEBC proceeds at a slightly faster rate than tone-CS-cued dEBC

**Fig 4:**
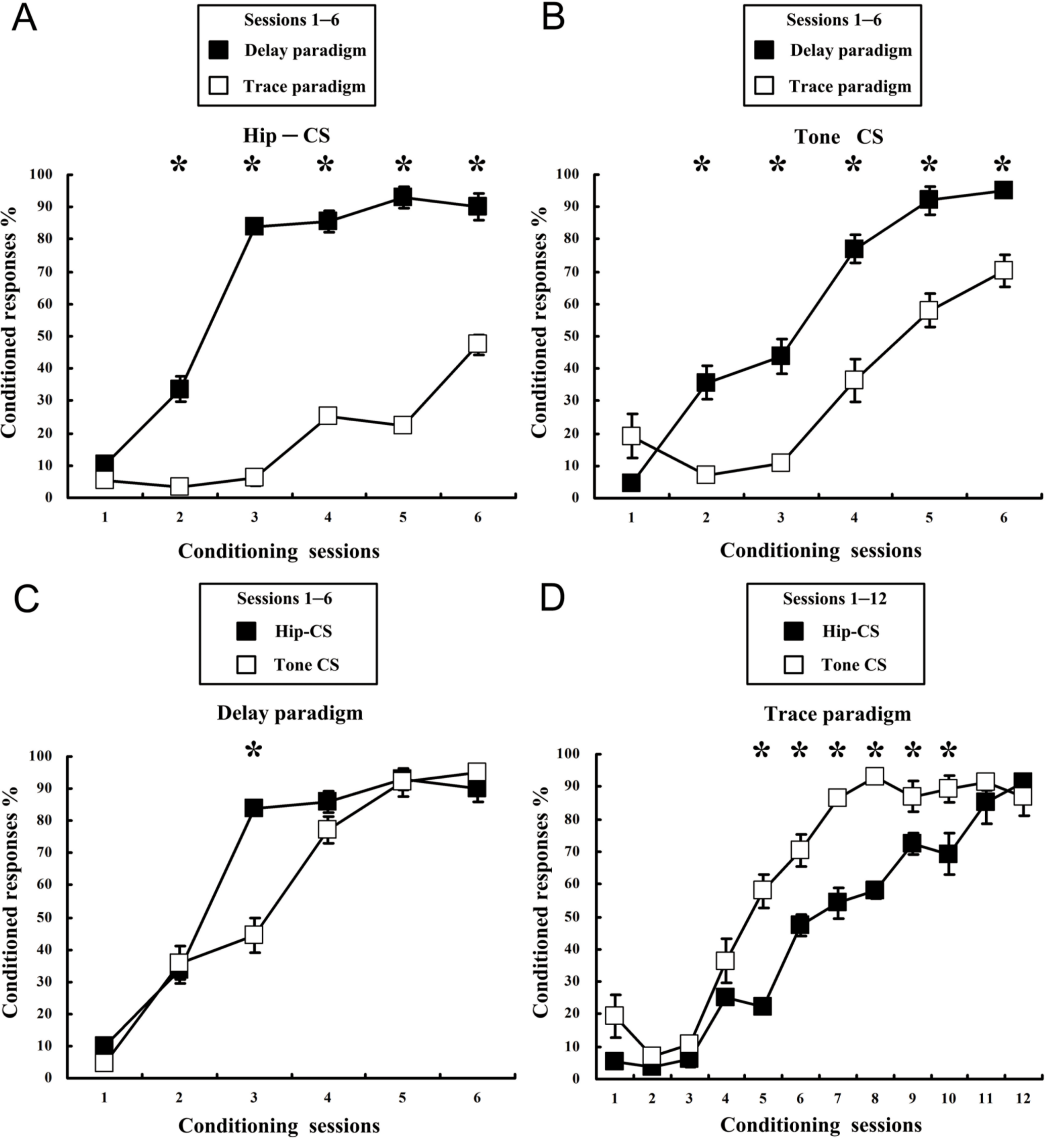
Comparisons of pre-shift CR acquisition rates between dEBC and tEBC, and between with Hip-CS and with tone-CS. Four curves depicting pre-shift CR acquisition rates in Fig 3 were rearranged and illustrated. Data represent mean ± SEM. (A, B), comparison of CR acquisition between paradigms of dEBC and tEBC. dEBC establishment (black square) showed higher acquisition rate than tEBC (space square), for both learning with Hip-CS (A, n = 6, for both dEBC and tEBC) and tone-CS (B, n = 7, for dEBC; n = 5, for tEBC), confirmed by statistically significant main effects of group [Fig 4A and 4F(1, 10) = 439.401, *p < 0.05; Fig 4B and 4F(1, 10) = 57.1, *p < 0.05], a two-way repeated measures ANOVA, followed by the LSD post hoc test; Only 6 sessions of data from trace paradigm are displayed (Fig 4A and 4B) to equal the time course with delay paradigm. (Fig 4C and 4D), comparison of CR acquisition between with Hip-CS and with tone-CS, across 6 or 12 training sessions (n = 6, for Hip-CS/dEBC; n = 7, for tone-CS/dEBC; n = 6, for Hip-CS/tEBC; n = 5, for tone-CS/tEBC). dEBC establishment showed higher acquisition rates when cued with Hip-CS (black square) than with tone-CS (space square), but tEBC establishment showed lower acquisition rates when cued with Hip-CS (black square) than with tone-CS (space square), confirmed by statistically significant main effects of group [Fig 4C and 4F(1, 11) = 5.635, *p < 0.05; Fig 4D and 4F(1, 9) = 70.117, *p < 0.05], a two-way repeated measures ANOVA, followed by the LSD post hoc test.

To compare the learning difference between the delay and trace paradigms in stage I to a given CS (Hip-CS or tone-CS), curves representing pre-shift CR acquisition rates in Fig 3 were rearranged accordingly and illustrated in Fig 4A and 4B. Comparison of CR acquisition cued with Hip-CS between dEBC and tEBC showed that animals acquired dEBC more rapidly than tEBC (Fig 4A), in agreement with the result of learning with tone-CS in this study (Fig 4B) and of other previous reports using tone or light stimulation as peripheral CS [40–42]. A two-way repeated measures ANOVA followed by the LSD post hoc test revealed significant main effects of group [Fig 4A and 4F(1, 10) = 439.401, p < 0.001; Fig 4B and 4F(1, 10) = 57.1, p < 0.001] and session [Fig 4A and 4F(5, 50) = 193.169, p < 0.001; Fig 4B and 4F(5, 50) = 81.498, p < 0.001]. Note that when comparing pre-shift CR acquisition difference between delay and trace paradigms, only 6 sessions of data from trace paradigm are displayed (Fig 4A and 4B) to equal the time course with delay paradigm.

To compare the effects of Hip-CS and tone-CS on CR establishment in the first training stage in the experiment (paired) groups, including both learning of dEBC and tEBC, the 4 curves depicting pre-shift CR acquisition rates in Fig 3 were rearranged and showed in Fig 4C and 4D. It was obvious that in dEBC, CR establishment with Hip-CS showed higher acquisition rates than with tone-CS (Fig 4C); but in tEBC, CR establishment with tone-CS showed higher acquisition rates than with Hip-CS (Fig 4D). There was significant main effects of group [Fig 4C and 4F(1, 11) = 5.635, p = 0.037; Fig 4D and 4F(1, 9) = 70.117, p < 0.001] and session [Fig 4C and 4F(5,55) = 237.939, p < 0.001; Fig 4D and 4F(11,99) = 111.013, p < 0.001].

### After CS shift, dEBC proceeds at a significantly faster rate than tEBC when cued with Hip-CS or tone-CS; Hip-CS-cued dEBC proceeds at a slightly faster rate than tone-CS-cued dEBC

**Fig 5:**
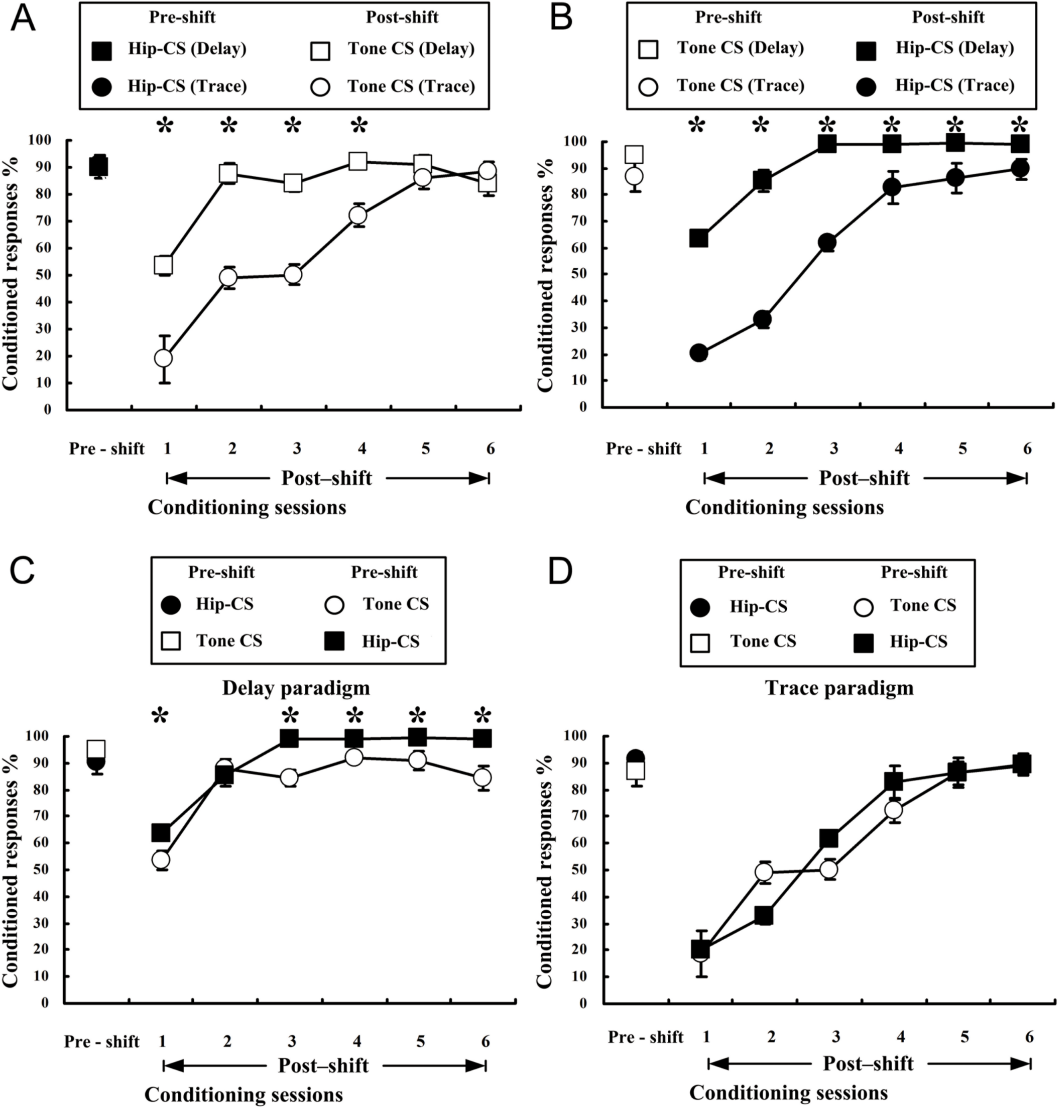
Comparisons of post-shift CR acquisition rates between dEBC and tEBC, and between with Hip-CS and with tone-CS. Four curves depicting post-shift CR acquisition rates in Fig 3 were rearranged and illustrated (for comparison, pre-shift CR acquisition rate in the last day of stage I was also demonstrated). Data represent mean ± SEM. (A, B) comparison of CR acquisition between paradigms of dEBC and tEBC (n = 6, for tone-CS/dEBC; n = 6, for tone-CS/tEBC; n = 7, for Hip-CS/dEBC; n = 5, for Hip-CS/tEBC). For post-shift learning with both tone-CS (A) and Hip-CS (B), establishment of dEBC (square, space or black) showed higher acquisition rates than of tEBC (roundness, space or black), confirmed by statistically significant main effects of group [Fig 5A and 5F(1, 10) = 53.918, *p < 0.05; Fig 5B and 5F(1, 10) = 92.772, *p < 0.05], a two-way repeated measures ANOVA, followed by the LSD post hoc test. (C, D) comparison of CR acquisition between with Hip-CS and with tone-CS (n = 6, for tone-CS/dEBC; n = 7, for hip-CS/dEBC; n = 6, for tone-CS/tEBC; n = 5, for Hip-CS/tEBC). Post-shift learning with Hip-CS (black square) showed significant difference relative to with tone-CS (space roundness) for establishment of dEBC (Fig 5C and 5F(1, 11) = 26.796, *p < 0.05), but not of tEBC (Fig 5D and 5F(1, 9) = 0.113, p = 0.745), confirmed by a two-way repeated measures ANOVA, followed by the LSD post hoc test.

To compare the learning difference between dEBC and tEBC in stage II to a given CS (tone-CS or Hip-CS), curves representing post-shift CR acquisition rates in Fig 3 were rearranged accordingly and illustrated in Fig 5A and 5B. Comparison of learning efficiency between delay and trace paradigms with tone-CS (Fig 5A) or Hip-CS (Fig 5B) was illustrated. There are significant main group effects in learning with tone-CS [Fig 5A and 5F(1, 10) = 53.918, p < 0.001] and with Hip-CS [Fig 5B and 5F(1, 10) = 92.772, p < 0.001].

To compare the difference between effects of central CS and peripheral CS on CR establishment after CS shift in experimental groups, including both delay and trace paradigms, the 4 curves depicting post-shift CR acquisition rates in Fig 3 were rearranged and showed in Fig 5C and 5D. Comparison of effects between Hip-CS and tone-CS on post-shift CR establishment showed significant main group effect in delay paradigm [Fig 5C and 5F(1, 11) = 26.796, p < 0.001], but not in trace paradigm [Fig 5D and 5F(1, 9) = 0.113, p = 0.745].

## Discussion

The present study has shown that microstimulation of the hippocampus as a CS paired with a US is sufficient to establish dEBC and tEBC in guinea pigs and that the establishment of tEBC with Hip-CS is slower than that of dEBC, which is in agreement with previous studies using peripheral CS (e.g., a tone or a light CS) to establish EBC [41, 42]. It has been well established that with peripheral CS, dEBC can be readily acquired, and it requires only the brainstem and cerebellar structures, whereas tEBC, or dEBC cued with low CS intensity, cannot be acquired without the involvement of additional brain sites, such as the hippocampus and mPFC, in addition to the abovementioned structures [35, 43–53].

In contrast to the commonly used auditory CS, which consists of acoustic stimulation with an intense 80–90 dB pure tone that always elicits significant startle reflexes (SR), the hippocampal microstimulation used in the present study was strictly controlled below 40% of the minimal current level capable of initiating a measurable UR. The electrophysiological recording demonstrated that mPFC stimulation with 200 μA or less did not evoke any field potentials in the motor cortex, somatosensory cortex, or the cerebellar cortex in the guinea pigs. This finding suggests that the hippocampus-induced EBC is established by direct stimulation of the hippocampus as a CS rather than stimulation of other brain regions as a CS.

Given that the hippocampus is typically necessary for tEBC [35, 43–45] but not for dEBC, which suggests the involvement of the hippocampus in a regulatory circuit for tEBC but not for dEBC, we expected that tEBC would be acquired more rapidly than dEBC when the Hip-CS was used. However, instead, the Hip-CS-induced tEBC was acquired more slowly than that of the Hip-CS-induced dEBC. Several studies have also demonstrated the similar unexpected results previously. For instance, stimulation of the anterior pretectal nucleus, an important region proved to be related to EBC acquisition by lesion experiments [54], did not serve as an effective CS when paired with a US to establish EBC [10]; whereas stimulation of the visual cortex, which is not involved in the acquisition of EBC [54], was successfully used as an effective CS when paired with a US to establish EBC [12]. It is possible that hippocampal stimulation actually interfered with the hippocampal processing required to establish and maintain trace conditioning.

In addition, for the tEBC establishment, our experiment showed that the central CS, hippocampal microstimulation, is much less effective than the peripheral CS, a tone. The potential mechanism for this difference is that tone signals are binaurally projected upstream and may activate distributed central structures, including the thalamic nuclei and inferior colliculus [55, 56], primary auditory cortex [57], and even the mPFC [58], while hippocampal microstimulation is only delivered to a localized locus within one hemisphere. However, it is more feasible to establish dEBC with a Hip-CS than with a tone-CS. The diversity in the effectiveness of the Hip-CS in inducing dEBC and tEBC may be derived from the following two possibilities. First, hippocampal stimulation may have interfered with the hippocampal processing required to establish and maintain tEBC but not dEBC. Second, differences between dEBC and tEBC in the functional association between the neural circuit mediating the specific behavior and the brain loci where electrical stimulation was applied. Given that there is no direct projection from the hippocampus to the pontine nucleus (PN), which is a key relay station for sending CS signals to the cerebellum, more research is needed to elucidate the indirect and possibly distributed connection between the hippocampus and the PN during the establishment of Hip-CS-induced EBC.

Our previous work indicated that exciting the mPFC with electrical microstimulation [26] or with optogenetics [59] as a CS (mPFC-CS) can successfully establish classical EBC, and that learning transfer is available between EBCs established with mPFC-CS and tone-CS [34]. The present study demonstrates that learning transfer is likely to occur between conditioned learning induced by Hip-CS and tone-CS in both the dEBC and tEBC paradigms, which is manifested by an immediate or slow increase in CR% to the second CS, on the first day after the CS shift, or on the successive days during the second training stage. Furthermore, transfer learning (from hippocampus to peripheral, or vice versa) does not interfere with recall of the original memory for the CS-US association in the first stage, which is consistent with Rocio Leal-Campanario’s research about the transfer of tEBC established with a central CS, primary somatosensory cortex stimulation, and a peripheral CS, tone [23].

During post-transfer training (stage II), the CR reacquisition rate was significantly higher in dEBC than in tEBC, with both types of CS shifting from the hippocampus to a tone and vice versa. Given that before the CS shift, the CR% of dEBC and tEBC exhibited similar levels on the last training day of stage I (6th day for dEBC and 12th day for tEBC), the result in stage II suggests that the saving effect was more evident in dEBC than tEBC, or learning transfer was more effective for the simple task than for the difficult task. One potential reason may be that the CS–US association was much stable and effective in dEBC and thus more likely to be engaged by the sudden switch of the CS than in tEBC. In addition, we found that for the dEBC task, the transfer effectiveness is lower when shifting the CS from central to peripheral than vice versa. This result is likely because the peripheral tone-CS in stage I induced a wider circuit to support EBC than that of the central Hip-CS and thus provided a more reliable basis for learning transfer [34].

In our previous work of studying EBC transfer with electrical stimulation of mPFC or tone as CS [34], we noticed that CR acquisition rates in animals who had previously received unpaired stimulations of mPFC-CS and US were slightly higher than that of naïve animals who had received no CS or US stimulation before. In this study, we also observed the similar phenomenon as shown in Fig 3A (e.g., the 7th session of Tone-CS vs. the 1st session of Hip-CS), suggesting the existence of the possible unspecific facilitating effects of the previous unpaired CS1 and US stimulation on the subsequent CS2–US paired learning. It is, therefore, reasonable to speculate that the possible latent inhibition effect resulting from the unpaired training may not be as strong as the possible facilitating effects elicited by either CS1 or US stimulation alone or combination of both of them, on the subsequent conditioning.

Little is known about the underlying mechanism of EBC transfer when the CS shifted from the hippocampus to a tone or vice versa. It has been reported that the cerebellum and associated brainstem structures are essential for transfer of EBC learning [6, 60–63]. For example, the plasticity of the IPN and pontine nuclei (PN) correlated with the cross-modal learning of classical EBC [63]. Kehoe et al. have contributed many pioneering studies to expand the understanding of the mechanism of associative learning transfer. By using connectionist network models, they reported that CS2 might benefit from learning-related changes inside the cerebellum induced by CS1 [64, 65]. In addition, it has also been noticed that amygdala, thalamus, inferior colliculus and PFC might also be implicated in cross-modal savings [66–68].

In conclusion, animals successfully acquired the specific CR through conditioned microstimulation of the hippocampus, a key brain region involved in tEBC regulation. EBC transfer occurs when CS shifts from the hippocampus to peripheral sources or vice versa. The memory of the CS–US association is less disturbed in dEBC than in tEBC by the sudden switch of CS after both transfer types. Moreover, EBC transfer was more effective in a simple task (dEBC) than in a difficult task (tEBC). The present result significantly expanded our knowledge of EBC and the functional neural circuit underlying EBC, and this finding is helpful for understanding the common mechanisms underlying conditioned reflexes as well as learning and memory.

## Abbreviations

CR: conditioned response
CS: conditioned stimulus
UR: unconditioned response
US: unconditioned stimulus
dEBC: delay eyeblink conditioning
tEBC: trace eyeblink conditioning
Hip: hippocampus
SEM: standard error of the mean
IPN: cerebellar interpositus nucleus
PN: pontine nuclei
